# Influence of cold plasma activation on fluoride uptake and demineralization of enamel

**DOI:** 10.1007/s00784-026-07167-1

**Published:** 2026-09-26

**Authors:** Alexandra Schmidt, Lukas Zingen, Sarah Barke, Bianca Rohland, Marcus Harms, Rinat Ortmann, Wolfgang Viöl, Annette Wiegand

**Affiliations:** 1 Department of Preventive Dentistry, Periodontology and Cariology, University Medical Center Göttingen, Robert-Koch-Strasse 40, Göttingen, 37075 Germany; 2 Faculty of Engineering and Health, HAWK University of Applied Science and Arts Göttingen, Von-Ossietzky-Strasse 99, Göttingen, 37085 Germany

**Keywords:** Cold plasma, Fluoridation, Demineralization, Enamel

## Abstract

**Objectives:**

This in vitro study investigated the influence of cold plasma activation time prior to 1.25% fluoride gel application on fluoride uptake and demineralization of enamel.

**Materials and methods:**

Human enamel specimens (*n* = 200) were either treated with a dielectric-barrier plasma discharge at a constant distance (d = 1 mm), pulse repetition frequency (f_1_ = 1 kHz), power (P_mean_=36 mW) and voltage (U_pp_=28 kV) for 0.5, 1, 2, or 30 s or received no plasma activation (each *n* = 40). Subsequently, half of the specimens from each group were coated with fluoride gel (1.25% NaF/AmF, t = 5 min). In each group, one subset of specimens (*n* = 10) was treated with KOH (1 M, 24 h) to quantify surface-deposited fluoride. Remaining specimens (*n* = 10) were demineralized (citric acid, pH 2.3, 60 s) to determine structurally bound fluoride as well as calcium and phosphate release. Statistical analysis was performed using linear mixed models and generalized linear mixed models with Tukey post-hoc tests (*p* < 0.05).

**Results:**

Fluoride gel significantly increased KOH-soluble and structurally bound fluoride and reduced calcium and phosphate release. Cold plasma activation had no significant effect on fluoride uptake or calcium and phosphate release. Plasma activation for 2 and 30 s resulted in a significant reduction of the calcium/phosphate ratio compared to the groups without plasma activation and without fluoride gel (*p*_adj_.<0.001).

**Conclusions:**

Single cold plasma activation prior to fluoride gel application did not improve fluoride uptake or reduce demineralization of enamel.

**Clinical relevance:**

Under the investigated conditions, additional cold plasma activation provided no benefit beyond conventional topical fluoride treatment.

## Introduction

The use of fluoride for the prevention of dental caries and the reduction of erosive tooth wear is well established. The preventive effect of fluoride is mainly attributed to its ability to inhibit enamel demineralization and enhance remineralization [1]. Topical fluoride application leads to the formation of calcium fluoride or CaF_2_-like precipitates on the enamel surface, which function both as a physical barrier and as a reservoir for sustained fluoride release. Furthermore, fluoride is incorporated in the enamel structure by forming fluorapatite, which is less susceptible to acid attacks than hydroxyapatite. A large amount of these calcium fluoride surface deposits is lost relatively quickly by mechanical removal and dissolution, so that it is necessary to repeat the topical fluoride application regularly [2, 3]. Increasing the beneficial effects of the topically applied fluoride or enhance the fluoride retention would be a great advantage and modification of the surface prior to fluoride application might be a promising approach, which has also been studied by laser treatment [4].

In the recent years cold plasma technology has gained interest as a noninvasive treatment method and has demonstrated promising effects in terms of sterilization, surface modification, and the enhancement of adhesion [5–7]. Nonthermal plasma contains photons, electrons, positive and negative ions, atoms, free radicals, and excited or nonexcited molecules. Their potential application is linked with the production of various reactive oxygen and nitrogen species (RONS) which interact with the substrate the plasma is applied on and can generate a more reactive surface [8, 9].

Initial studies examining the combined application of plasma treatment and fluoride gels or varnishes indicate that this may enhance fluoride uptake or retention in hydroxyapatite [10] and enamel [11–13] compared to the application of fluoride gel alone. In those studies, the effects of different plasma sources have been compared but no systematically evaluation of the impact of individual treatment parameters, particularly plasma application time was done. Consequently, there is a lack of evidence-based guidance regarding the optimal parameters of the combined treatment of cold plasma activation and fluoride agents. The present study addresses this by systematically varying plasma application times and assessing their effects on topically deposited and structurally bound fluoride in enamel. In addition, the release of calcium and phosphate after demineralization, as well as the resulting calcium/phosphate ratio, were evaluated.

The null hypothesis was that variation of the cold plasma application time has no significant effect on fluoride uptake and enamel demineralization.

## Materials and methods

This study was approved by the local ethics committee of the University Medical Center Göttingen (approval number 27/8/24).

### Study design

To systematically investigate the effect of varying plasma application times on fluoride uptake as well as calcium and phosphate release from dental enamel, a multi-stage experimental design was developed. In each experimental group (*n* = 40), dielectric barrier discharge plasma was applied for 0.5 s, 1 s, 2 s, or 30 s. No cold plasma treatment was applied in the control group (*n* = 40). Subsequently, half of the specimens of each group received an application of 1.25% fluoride gel for 5 min. Thereafter, in each group, a subset of specimens (*n* = 10) was used to determine surface-bound fluoride. The remaining specimens (*n* = 10) were subjected to enamel demineralization to assess structurally bound fluoride. In addition, the demineralization resistance was tested by determination of the concentrations of dissolved calcium and phosphate in the demineralization solution, and furthermore the calcium/phosphate ratio was calculated.

### Specimen preparation

Cylindrical enamel specimens were prepared from caries-free, intact human third molars using diamond-coated trepan burs (custom-made, Gebr. Brasseler) and embedded in resin (Paladur, Kulzer). A defined layer of 400 μm was removed from the enamel surface of each specimen, and the surfaces were polished using water-cooled abrasive papers (WS flex 18 C, grit 1,200, Hermes; silicon carbide, grit 4,000, Buehler) with a microgrinding system (400 CS, Exakt, Norderstedt, Germany). Specimens exhibiting microscopically visible irregularities or cracks were discarded.

From each tooth, two specimens were obtained and used matched in one of the subgroups with identical plasma treatment time. One specimen of each pair was treated with fluoride gel, while the other remained untreated. The specimens were stored in deionized water until further use.

### Cold plasma application

**Fig. 1:**
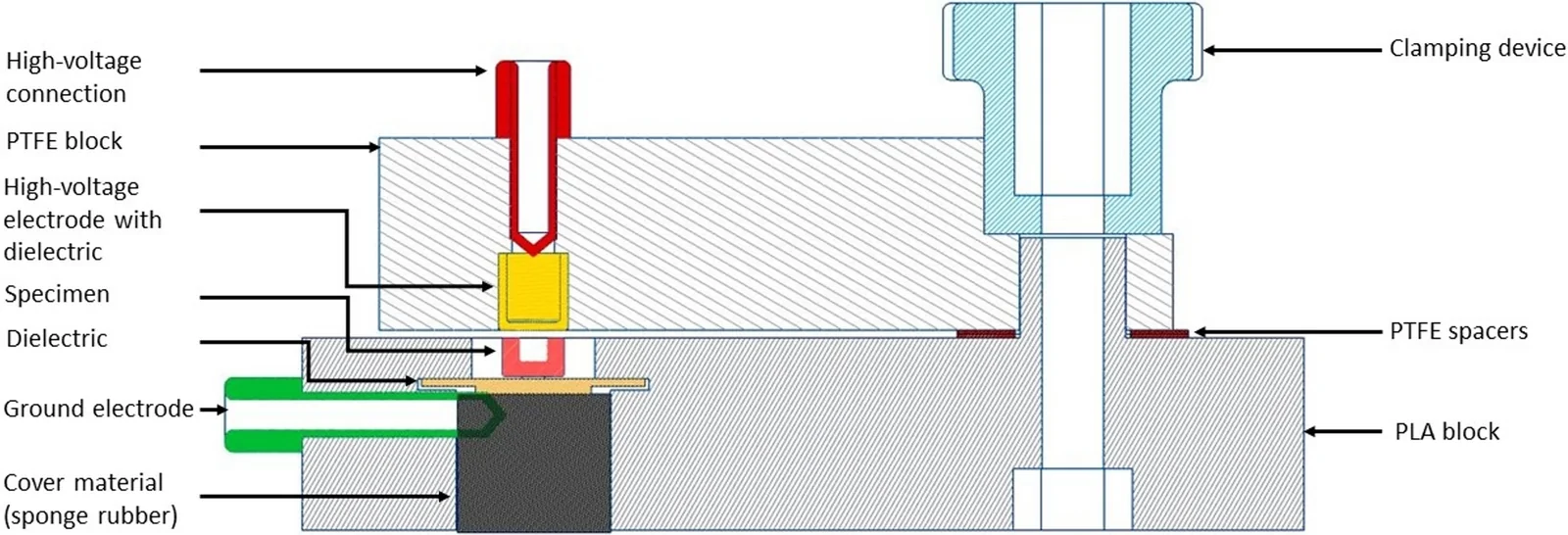
Schematic design of the plasma source

Enamel specimens were treated with cold plasma at four different application times, t_1_= 0.5 s; t_2_= 1 s; t_3_= 2 s; t_4_= 30 s, at a constant distance of 1 mm, pulse repetition frequency (f_1_ = 1 kHz), pulse eigenfrequency (f_2_ = 196 kHz), power (P_mean_ = 36 mW) and voltage (U_pp_ = 28 kV). Electrical power measurements were performed using the UI method [14]. All work processes were monitored using an oscilloscope (YOKOGAWA DLM 2054, 2.5 GS/s, 500 MHz, Yokogawa Meters & Instrument Corporation, Tokyo, Japan). A high-voltage probe (Tektronix P6015A, Tektronix Inc., Beaverton, USA) was used to measure the applied voltage. The plasma source was custom-built for this project (Fig. 1).

The main components of the plasma source were a high-voltage block and a grounded block, in which the test specimen chamber was positioned. The upper part contained a high-voltage generator fixed in a PTFE block for the plasma generation. The electrode was surrounded by an aluminum oxide to stabilize the generated plasma. Directly below the electrode was the specimen chamber allocated in PLA block. The top of the chamber was insulated with a foam rubber covering material to focus the plasma and minimize environmental influences. The dielectric was positioned under the test chamber and accompanied by a grounded electrode, which was electrically connected via a ground connection. This arrangement allowed the generation of a controlled electric field to ensure the gas ionization required for the formation of cold plasma. Two thin PTFE spacers (each 0.5 mm high) were inserted between the clamping device. These ensured a constant distance between the plasma source and specimen throughout the entire plasma treatment period. Each test specimen was leveled in the test chamber with modeling clay before the plasma application to ensure an even plasma discharge on the enamel surface.

### Fluoride gel application

For the topical fluoride treatment of the enamel surface a 1.25% fluoride gel (ELMEX gel, LOT number: 3173FR63B3, CP GABA GmbH, Hamburg, Germany) was used, which contains olafluor, dectafluor and sodium fluoride as well as purified water, propylene glycol, hydroxyethylcellulose, saccharin, apple, peppermint, menthone flavorings and spearmint oil. Each test specimen was coated with approximately 3 mg of fluoride gel using microapplicators (normal blue, Henry Schein, Langen, Germany). After an exposure time of 5 min, the fluoride gel was removed by rinsing with deionized water for 60 s. Subsequently the test specimens were stored for 1 h in 1 ml of artificial saliva [15, 16]. Thereafter, the specimens were removed, rinsed with deionized water for 30 s, dried, and subjected to further treatment.

### Demineralization

To determine the structurally bound fluoride and assess demineralization resistance, the enamel surface of respective test specimen was demineralized by adding 700 µl of 0.0025 M citric acid (pH 2.3). After incubation for 60 s under continues agitation the reaction was terminated by adding 350 µl of 0.1 M NaOH solution. The specimens were then removed, and the remaining solution was analyzed for fluoride, calcium and phosphate concentrations.

### Fluoride analysis

For the analysis of KOH soluble and structurally bound fluoride content, the respective test solution was mixed 1:1 with TISAB II solution (AnalytiChem GmbH, Duisburg, Germany) to avoid influence of interfering ions. The fluoride content was determined using a fluoride electrode (Electrode F 800 Din, WTW, Weilheim, Germany) on the inolab pH/ION 7320P (WTW, Xylem Analytics GmbH, Germany). Calibration was performed using three standards in the concentration range of 0.01-1 mg/l, prepared from a fluoride standard solution (10 g/l sodium fluoride from WTW, Xylem Analytics Germany). The test solutions were measured in duplicate and the results were normalized to the surface area of the respective test specimen. For surface area measurement a digital microscope was used (Smartzoom 5, PlanApo D 1.6×/0.1 FWD 36 mm objective; software version 1.4; firmware version 0.3.0-642; Zeiss, Jena, Germany). Surface area measurements were performed by applying a color threshold using Fiji image analysis software (version 1.54p).

To dissolve the KOH soluble fluoride the method according to Caslavska et al. was used [17]. 500 µl of 1 M KOH were added to the respective test specimen and stored on a shaker (Unimax 1010, Heidolph Instruments, Schwabach, Germany) under constant movement. After 24 h, KOH was neutralized with 500 µl 1 M HNO_3_ and the test specimen was removed. The fluoride content of the remaining solution was determined, and the amount of KOH soluble fluoride (F_KOH_.) was determined individually for each test specimen in ng/mm².

The structurally bound fluoride content (F_struct_.) was determined from the demineralization solution in ng/mm² for each test specimen.

### Calcium analysis

The calcium content was determined using the Arsenazo III method (Colorimetric Calcium AS FS Kit, REF 1 1130 99 10 021, Diagnostic Systems GmbH, Holzheim, Germany). The Arsenazo III method was used in accordance with the manufacturer’s protocol. Twenty microliters of the test specimen solution were mixed with 100 µl of calcium reagent, and after incubation for 5 min at room temperature, the absorption was measured at a wavelength of λ = 650 nm using a plate photometer (Spectra max M2, software SoftMax Pro 4.8, Molecular Devices, San Jose, California, USA). Since calcium forms a chelate complex with citric acid, that might mask calcium ions this was taken into account by a matrix matching approach by adding an equivalent amount of citric acid to the standard calibration series [18].

### Phosphate analysis

The phosphate analysis was based on the malachite green procedure (Colorimetric Phosphate Assay Kit, AB 65622, abcam, Cambridge, England). 30 µl of the phosphate reagent were added to 200 µl of test solution and, after incubation for 30 min at room temperature in the dark, absorption was measured at λ = 650 nm using a plate photometer (Spectra max M2, software SoftMax Pro 4.8, Molecular Devices, San Jose, California, USA).

### Statistical analysis

Statistical analysis was performed using R (R Core Team, 2021; version 4.4.3). Data distributions were assessed using Q–Q plots. Calcium and phosphate levels and the calcium/phosphate ratio were normally distributed, whereas deviations from normality were observed for the KOH-soluble and structurally bound fluoride. To evaluate the effects of plasma treatment time and fluoride application on calcium, phosphate, and the calcium–phosphate ratio, linear mixed-effects models (LMMs) were applied. Due to the non-normal distribution of the fluoride parameters, generalized linear mixed-effects models (GLMMs) were used. Post hoc tests with Tukey’s p-value adjustment were conducted for pairwise group comparisons. The level of significance was set at α = 0.05 for all analyses.

## Results

A total of 200 test specimens were examined and evaluated in accordance with the study design. Five parameters were analyzed in each study group: KOH soluble fluoride (F_KOH_), structurally bound fluoride (F_struct_), calcium (Ca), phosphate (P), and the calcium-phosphate ratio (Ca/P ratio). The descriptive values (mean ± SD) of the test groups are shown in Table 1.

**Table 1:** Mean values of on the surface deposited and structurally bound fluoride, calcium, phosphate and Ca/PO_4_ ratio. Superscript letters indicate statistically differences of values within a column (Tukey post hoc test, *p* < 0.05)

Plasma	Fluoride gel	F_KOH_	F_struct_	Ca	PO_4_	Ca/PO_4_ ratio
(s)	(min)	(ng/mm²)	(ng/mm²)	(nmol/mm²)	(nmol/mm²)	
-	-	5.4 ± 1.1^a^	3.8 ± 0.9^a^	49.2 ± 7.2 ^a^	25.8 ± 3.5 ^a^	1.9 ± 0.2 ^a^
	5	308.8 ± 98.3^b^	15.2 ± 4.2 ^b^	30.5 ± 9.9 ^b^	17.1 ± 6.3 ^b^	1.8 ± 0.2 ^a^
0,5	-	5.5 ± 1.1 ^a^	3.4 ± 0.8 ^a^	49.6 ± 13.0 ^a^	28.8 ± 9.4 ^a^	1.8 ± 0.3 ^a^
	5	287.8 ± 47.8 ^b^	17.9 ± 4.9 ^b^	36 ± 10.6 ^b^	20.7 ± 6.1 ^b^	1.8 ± 0.2 ^a^
1	-	5.6 ± 0.9 ^a^	3.4 ± 0.8 ^a^	47.4 ± 9.4 ^a^	27.9 ± 5.9 ^a^	1.7 ± 0.2 ^a^
	5	269.4 ± 65.7 ^b^	17.3 ± 7.7 ^b^	34.3 ± 12.2 ^b^	19 ± 5.9 ^b^	1.8 ± 0.1 ^a^
2	-	5.2 ± 1.1 ^a^	3.9 ± 1.2 ^a^	55.1 ± 12.1 ^a^	34.4 ± 8.5 ^a^	1.6 ± 0.2 ^b^
	5	288.8 ± 76.5 ^b^	16.9 ± 7 ^b^	33.7 ± 15.7 ^b^	19.9 ± 11 ^b^	1.8 ± 0.3^a^
30	-	5.2 ± 1.2 ^a^	3.1 ± 0.8 ^a^	48.1 ± 7.3 ^a^	31.2 ± 5.7 ^a^	1.6 ± 0.2 ^b^
	5	242.6 ± 81.4 ^b^	17.7 ± 8.9 ^b^	26.9 ± 9.2 ^b^	17.4 ± 6.4 ^b^	1.6 ± 0.1 ^a^

For all ion analysis a statistic significant influence from topical fluoride treatment was found, increasing the amount of fluoride and decreasing the calcium and phosphate release. Plasma treatment generated no statistically significant changes on the ion levels. Neither the variation in plasma treatment time nor the combination effect between plasma and fluoridation showed a statistically significant influence.

The mean Ca/P ratio of the non-fluoridated and non-plasma-treated specimens in the negative control group was 1.9 ± 0.2. Short plasma treatment durations (0.5 to 2 s) resulted in values ranging from 1.6 to 1.8, whereas a treatment time of 30 s led to a further decrease, with a mean Ca/P ratio of 1.6 ± 0.2. A statistically significant effect of plasma treatment time on the Ca/P ratio was found without fluoridation between the 30 s and 2 s group compared to the control group (*p* < 0.01). No statistically significant interaction effect between plasma treatment and fluoridation, nor a significant main effect of fluoridation alone, was observed on the Ca/P ratio.

## Discussion

This study investigated the effect of cold plasma activation combined with fluoride gel application on the fluoride uptake and demineralization resistance of enamel. Fluoride gel, but not cold plasma application, affected fluoride uptake and demineralization resistance. As the additional cold plasma activation had no significant effect on the fluoride uptake and demineralization resistance the null hypothesis was confirmed.

These results are in contrast to recent studies, which show a positive effect on the uptake or retention of fluoride by combining cold plasma activation with the use of topical fluoride agents [10–13]. The beneficial uptake of fluoride by combining plasma application with the use of fluoride agents is very sensitive to the properties of plasma source. Fathollah et al. [13] compared three plasma jets with different carrier gas - only one, a He-gas jet has shown a significant effect on the fluoride uptake. When using an Ar-Jet or Air-Jet plasma no influence on the fluoride uptake was found. The different fluoride uptake was attributed to the varying masses of the carrier gases used. The proposed mechanism suggests that heavier carrier gas particles transfer greater momentum to the enamel surface causing smoothing of surface irregularities. In contrast lighter carrier gas particles may induce smaller pits resulting in an increased surface roughness and thereby in a higher active surface area, which could enhance fluoride adsorption [13].

Furthermore, the frequency of the used plasma jet has an influence on the fluoride uptake, showing increasing fluoride levels with higher frequency. While almost all studies analyzing the effect of plasma treatment on fluoride uptake used plasma jets with a high frequency of 10–50 kHz, the present study used a dielectric barrier discharge plasma source, which generally operate at a lower frequency, here with 1 kHz. As no plasma jet was used, there was only ambient air present and no additional gas flow accompanied with the plasma application. In regards to this the distance between plasma source and enamel surface was kept short at 1 mm in contrast to up to 1 cm when using a plasma jet, to reduce loss of reactive species and directly ignite the plasma at the enamel surface. The dielectric barrier discharge plasma source used in this study was custom-built with the premise of a simple and easy to use design, which is cost saving as no noble gas is needed for application. It should also have the potential to be upscaled, so that the plasma can be applied to a larger surface area and is not limited to the area of the jet.

The plasma application times of 0.5 to 30 s are much lower than the 3 min treatment time in other studies [10, 11, 13], which goes hand in hand with a shorter reaction time of reactive species with the enamel surface. Clinically, a 3 min treatment time is rather long, especially when considering that only a small area is treated at one time, so that shorter treatment times would be beneficial. It was shown that also short treatment times around 1 s can lead to a modification of the enamel surface [19].

The exact mechanism by which cold plasma affects enamel has not yet been fully resolved. Proposed effects include increased surface wettability due to reduced contact angle and the etching of tooth surface, which may contribute to enhanced fluoride uptake into enamel [20]. Those observed effects are attributed to the action of reactive species, including ionized and neutral molecules as well as free radicals [5, 19]. Precise quantification of individual reactive species and determination of the effective plasma dose are challenging. A commonly applied approach involves the measurement of ozone, oxygen, and nitrogen species concentrations [21]. However, these species exhibit strong process-dependent behavior and are difficult to quantify, as their recombination times range from nanoseconds to microseconds. Due to the confinement of the plasma within the source and the dependence of plasma intensity on the substrate, direct measurement of the plasma interacting with the specimen surface is virtually impossible. Plasma characterization is commonly achieved by controlling the electrical parameters of the plasma source. In the present study, this was done by monitoring and maintaining constant voltage, current, and frequency throughout the entire experimental period.

Besides the different operating principles of the cold plasma activation devices, other studies applied a cyclic treatment protocol rather than a single plasma application. Also sequential treatment protocols have been used, where plasma has been applied before and after fluoridation [11–13]. The beneficial effects of plasma application in those studies on the fluoride uptake increased with the number of cold plasma activation times. With a single plasma treatment, no or only low increase of the fluoride uptake was found, which is in accordance to the study of Khoubrouypak et al. where no significant influence on the enamel resistance through combined application of cold plasma activation and fluoride varnish could be found [22]. Generally, cold plasma application is considered to be noninvasive [7]. However, the in the plasma created reactive species interact with the surrounding environment. This should be considered when applying plasma after topical fluoride application, as harmful reaction products in the fluoride gel or varnish could be generated. Zarif et al. reported staining when applying plasma after coating with fluoride gel [10]. To avoid potential generated toxic side products this study did not include plasma treatment after fluoride gel application. In general, plasma sources for dental surface treatment are currently designed for use by dental professionals rather than for home use. Therefore, a single plasma treatment better reflects the clinical setting than repeated treatment.

The demineralization resistance of enamel was determined by analyzing photometrically the calcium and phosphate release into citric acid. As plasma activation is mainly a surface-related effect the demineralization time was kept short to limit demineralization to enamel surface layer and thereby avoid masking the potential effect of plasma activation by deeper demineralization. When using citric acid, it is important to note that it forms a chelate complex with calcium, which distorts the color-dependent measurement by masking calcium ions. To take the influence of masked calcium into account a matrix matching approach was used [18] by adding an equivalent amount of citric acid to the standard series. Furthermore Attin et al. has shown that the effect of chelate complex formation in calcium determination is reliable, if not very small amounts of calcium are being measured [23].

As expected the application of fluoride gel increased the demineralization resistance of enamel. Furthermore, an increase of calcium/phosphate ratio is expected originating from the precipitated calcium fluoride compounds and fluorapatite [3]. However, fluoridation did not affect the calcium/phosphate ratio in the present study. The estimated calcium/phosphate ratios are in accordance to literature and are close to the stochiometric value of 1.67 of hydroxyapatite [24]. In the non-fluoridated groups, a statistically significant decrease in the Ca/P ratio was observed at plasma treatment times of 2 s and 30 s compared with the control group. However, as no significant changes were detected in the absolute calcium and phosphate levels, these findings cannot be directly associated with an effect on demineralization resistance.

## Conclusion

Single cold plasma activation prior to the application of a fluoride gel did not improve fluoride uptake or reduce the demineralization of enamel. The application of fluoride gel significantly increased fluoride uptake and reduced calcium and phosphate release, confirming the protection against acid induced demineralization. The absence of an additional effect of plasma activation suggests that under the conditions of the present study - using the specific plasma source and treatment parameters in this study - a single short plasma activation is not able to enhance the effect of topical fluoride treatment.

## Data Availability

The datasets of the study are available from the corresponding author on reasonable request.
